# Physiology and pharmacological targeting of phase separation

**DOI:** 10.1186/s12929-024-00993-z

**Published:** 2024-01-20

**Authors:** Fangfang Wang, Youwei Zhang

**Affiliations:** Department of Pharmacology, School of Medicine, Case Comprehensive Cancer Center, Case Western Reserve University, 2109 Adelbert Road, W309A, Cleveland, OH 44106 USA

**Keywords:** Liquid–liquid phase separation (LLPS), Condensates, Inclusion bodies, Pathogenic LLPS, Targeting LLPS

## Abstract

Liquid–liquid phase separation (LLPS) in biology describes a process by which proteins form membraneless condensates within a cellular compartment when conditions are met, including the concentration and posttranslational modifications of the protein components, the condition of the aqueous solution (pH, ionic strength, pressure, and temperature), and the existence of assisting factors (such as RNAs or other proteins). In these supramolecular liquid droplet-like inclusion bodies, molecules are held together through weak intermolecular and/or intramolecular interactions. With the aid of LLPS, cells can assemble functional sub-units within a given cellular compartment by enriching or excluding specific factors, modulating cellular function, and rapidly responding to environmental or physiological cues. Hence, LLPS is emerging as an important means to regulate biology and physiology. Yet, excessive inclusion body formation by, for instance, higher-than-normal concentrations or mutant forms of the protein components could result in the conversion from dynamic liquid condensates into more rigid gel- or solid-like aggregates, leading to the disruption of the organelle’s function followed by the development of human disorders like neurodegenerative diseases. In summary, well-controlled formation and de-formation of LLPS is critical for normal biology and physiology from single cells to individual organisms, whereas abnormal LLPS is involved in the pathophysiology of human diseases. In turn, targeting these aggregates or their formation represents a promising approach in treating diseases driven by abnormal LLPS including those neurodegenerative diseases that lack effective therapies.

## Background

**Fig. 1:**
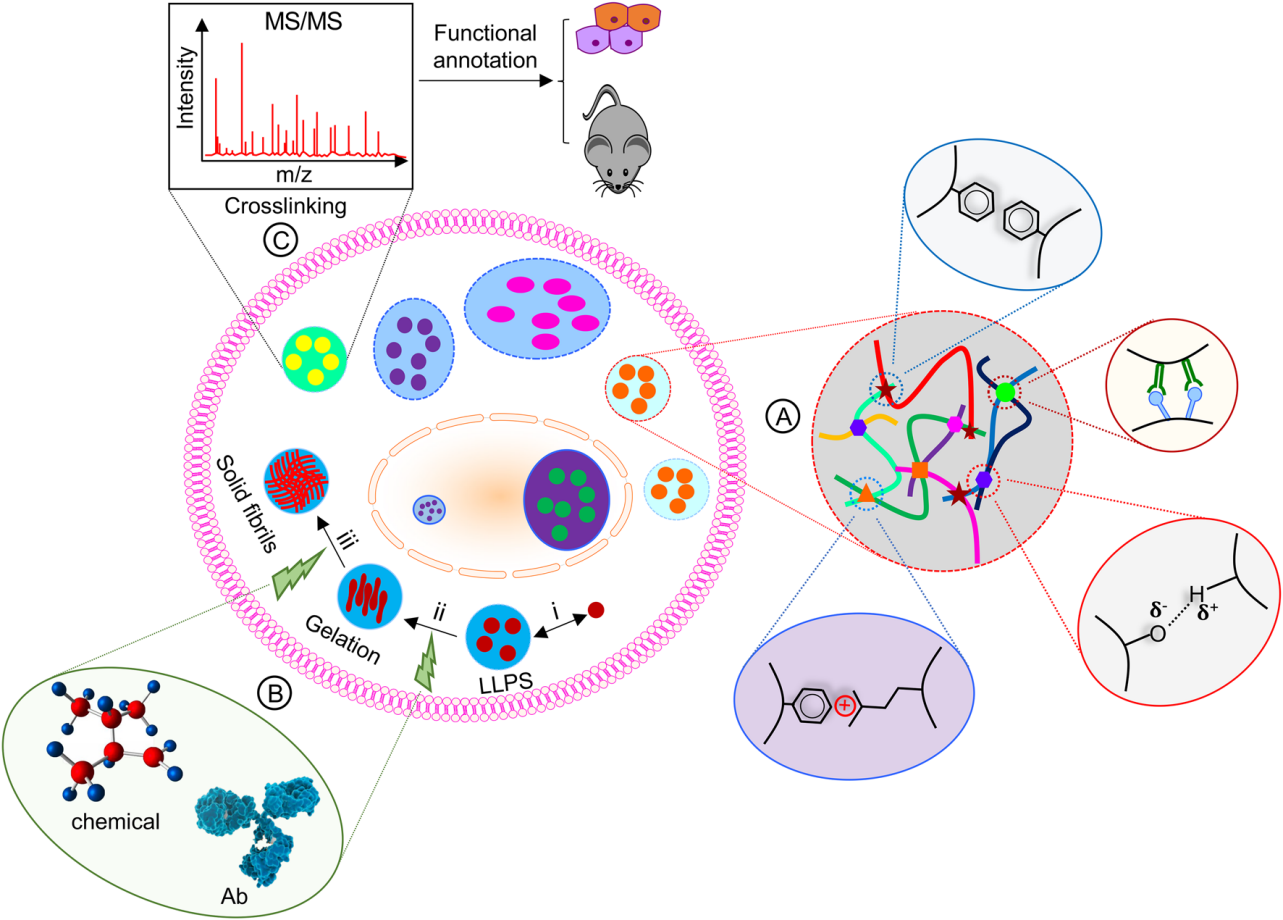
Biological assembly and function of LLPS both in the cytoplasm and in the nucleus. (**A**) The forces and interaction modules that promote LLPS. (**B**) Strategies that can target the pathogenic LLPS process by small molecules to block/slow/reverse the liquid–solid transition, or biological approaches such as monoclonal antibodies that can recognize the formed aggregates with unique structures, leading to amelioration of the disease. (i-iii) Representative illustration of the process showing the conversion from liquid droplets to gel- or solid-like aggregates during disease progression. While stage i is reversible, stages ii-iii are likely irreversible, leading to the formation of amyloid fibrils that are seen in patients with neurodegenerative diseases. (C) Determination of components in liquid droplets through mass spectrometry. An initial crosslinking step is suggested to stabilize the inter-molecular interactions within the liquid droplets, allowing  a high percentage of recovery of LLPS components for subsequent mass spec identification. Further functional analysis can be carried out in cell cultures and in animal models

The idea of liquid–liquid phase separation (LLPS) has long been recognized and studied in the polymer sciences field [1]. However, it was not until recently that LLPS was realized to also regulate biology, firstly reported in regulating P granules in *Caenorhabditis elegans* germline cells in 2009 [2]. In just slightly over a decade, LLPS is now recognized as an important biological means to regulate a wide variety of cellular functions. The most significant feature of LLPS in biological sciences is the appearance of membraneless organelles formed within cells, which is in sharp contrast to those conventional sub-cellular compartments like the lysosomes, the nucleus, the Golgi apparatus, and the endoplasmic reticulum that all contain a lipid bilayer membrane to embrace the contents within. The largest membraneless organelle in eukaryotic cells is the nucleolus, which is known to be scarce in DNA and the realization of its existence can be dated back to 1896 [3]; yet it wasn’t until 2011 that nucleoli were demonstrated to bear LLPS properties [4]. Currently, LLPS has been reported to contribute to a growing list of cellular sub-compartments, including the Cajal bodies, the paraspeckles, the protein/RNA ribonucleoprotein (RNP) bodies, and heterochromatin found in the nucleus, as well as germ granules, stress granules, and processing bodies (P-bodies) formed in the cytoplasm [5, 6] (Fig. 1). The LLPS concept has transformed our understanding of cellular information processing. Here, we will summarize recent advancements in our understanding of LLPS with a focus on its biological implications and potential targeting strategies. We will also discuss challenges in the field and provide our perspectives to address them.

### Physical appearance and properties of liquid droplets

Biological LLPS results in the formation of membraneless liquid droplets, condensates, or inclusion bodies by proteins and often with the aid of nucleic acids (such as RNA), through which it compartmentalizes the intracellular contents in a particular space and therefore affecting cellular and biological outcomes [7]. These condensates/liquid droplets can form over a broad spatial and temporal range, with the size spanning from nm to μm and the timescale from sub-seconds to even hours [8], which perfectly reflect the diverse involvement and function of LLPS in biology and physiology. Hence, LLPS provides a unprecedented biophysical basis to shape many cellular functions, such as gene expression and cell division [5, 9, 10], heterochromatin compaction [11–17], stress granule formation [18], mRNA splicing [19, 20], super-enhancer activity [21, 22], receptor- and non-receptor-modulated signal transduction at the cytoplasmic membrane [9, 23, 24], etc.

Most liquid droplets are displayed as circular condensates with a uniform phase, especially those formed in vitro by purified proteins. However, some can form multiple phases within a single droplet [10, 25], or even vesicle-like hollow spheres as recently presented by others and us for germ granules of *Drosophila* [26, 27], transactive response DNA binding protein of 43 kDa (TDP-43) [28], RNA-RNPs [29, 30] and TP53-binding protein 1 (53BP1) at heterochromatin [11]. For those aggregates that form along cellular membranes [9, 23, 31] or around heterochromatin [11], they may display non-circular irregular shapes. The shape and the size of liquid droplets are likely dependent on the tropism and electrostatic force empowered by the protein–protein and/or protein-nuclei acid complexes within the liquid droplets/condensates, as well as the physical force (like the surface tension) between the liquid droplets and the surrounding cellular environment.

On the other hand, the compositions of liquid droplets vary dramatically from a few [32, 33] to hundreds of thousands of proteins [34–37]. Yet, what determines the scale and the number of components in any given droplet assembly does not seem to follow a common rule, representing an ongoing research direction. It is tempting to speculate that the specific composition of a particular type of liquid droplets relies on the properties of both the core components, the so-called scaffold proteins [38], and the passengers that support the inclusion and exclusion of other factors. To facilitate the understanding of the biology of LLPS, a list of key terms that are important for describing and understanding the LLPS process is summarized in Table 1. Other terms related to physicochemical analysis of LLPS can be found in recent reviews [6, 8, 39].

**Table 1:** Main terms used to describe LLPS

Name	Description
Liquid droplets, condensates, or inclusion bodies	Terms describing the formation of membraneless organelles inside cells that are undergoing phase separation. These terms are used almost interchangeably in the literature when referring to the liquid state of the organelles. However, unlike liquid droplets, condensates and inclusion bodies could be gel- or solid-like proteinaceous structures that are involved in pathophysiology. In addition, condensates and inclusion bodies may adopt irregular shapes unlike the often round liquid droplets
IDR (intrinsically disordered regions)	Peptide regions in proteins that can drive/promote condensate formation in vitro and in vivo*,* which was firstly proposed by Michael Rosen’s lab in 2012
LCD (low complexity domains)	Domains found in proteins that can facilitate LLPS formation. LCD is made up of a particular subset of amino acids (like polar and charged residues, but less likely bulky hydrophobic residues) and tends to be intrinsically disordered in terms of the structure. Both IDR and LCD are considered floppy portions of proteins that do not form a stable structure but allow easy access of solvent or other interacting partners including proteins and RNAs. Further, IDR and LCD appear to serve as acceptors for protein post-translational modifications, which could enhance or reduce their ability in LLPS in a context-dependent manner
Weak molecule–molecule interaction	Non-covalent weak interactions among macromolecules (protein-nucleic acid, protein–protein, and nucleic acid-nucleic acid) for LLPS formation
Sticker	Short regions in IDR, LCD, or other structural domains such as oligomerization domains that are key for liquid droplets formation
Spacer	Flexible peptide sequences that arrange the spatiotemporal organization and interaction of ‘Stickers’
Scaffold	The major component(s) in a liquid droplet that drives the assembly/formation of the condensate/inclusion body
Client	The passengers in the liquid droplets, which do not initiate the liquid droplet formation, but contribute to the dynamics and the balance of formed inclusion bodies
Valency	The number of weak interactions that a molecule can provide to recruit other molecules, which also indicates the strength/ability of proteins and/or nucleic acids to form liquid condensates. The higher the valency, the easier it is for the components to form liquid condensates. Valency is affected by the presence of IDRs and LCDs, as well as surrounding solutions
Viscosity and viscoelasticity	Viscosity can be simply understood as the stickiness of a solution/condensate, through which it increases the resistance of the solution to free flow. Viscoelasticity describes the ability of the condensates to return to the pre-defined form after the removal of external stress/pressure

### LLPS regulates the genome structure and function

Our appreciation of phase separation in biology started from its role in stress granule regulation [2], which is now expanded into virtually every biological process and cellular function. While comprehensive reviews have been published recently [40], we will highlight recent discoveries that advanced the field with a particular focus on the role of LLPS in regulating nuclear chromosome organization and transcription. LLPS can promote the activation of the adaptive T-cell immunity through condensate formation of factors involved in the T-cell receptor (TCR) signaling while excluding the negative factor CD45 from the condensates [23]. LLPS also regulates the innate immunity, which involves phase separation of the cyclic GMP-AMP synthase (cGAS) with cytosolic DNA [41]. Pre- and post-synaptic neuron plasticity rely on condensate formation and phase separation of pre- and post-synaptic density proteins RIM/RIM-BP and PSD95/SynGAP, respectively, [42–44]. During autophagy, phase separation plays an important role in the formation of autophagosomes by Atg1 in yeast [45, 46] and SQSTM1/P62 in vertebrates [47, 48]. Phase separation of the type I subunit of protein kinase A (PKA RIα) mediates the homeostasis of intracellular 3’,5’-cyclic adenosine monophosphate (cAMP) and activation of the G protein coupled receptors; PKA RIα LLPS defect promotes cell growth and transformation [49].

Nuclear liquid condensates have attracted increasing awareness and attention due to their roles in directly interacting with and regulating the genome [5] through forming inclusion bodies at both transcriptionally active (like micrometer-sized nucleoli and super-enhancer regions) and less active (such as heterochromatin) domains, facilitating our understanding of chromatin structural organization and genetic information flow. Although the way by which these droplets regulate the genome function depends on the specific genomic environment, a very likely scenario is that LLPS regulates the chromosomal architecture and its accessibility to regulatory factors [50, 51], and thereby transcriptional regulation of genes with specific biological functions [50–53].

As the other side of the coin, transcriptional repression is as important as transcriptional activation in regulating the genome function. LLPS has been recently reported to mediate the repressive state of heterochromatin and therefore transcriptional silencing. The heterochromatin protein (HP1α) from human, flies and yeast formed liquid droplets when phosphorylated at the N-terminus or with the addition of DNA [12], although the mouse counterpart was a weak LLPS factor at best [54]. Other heterochromatin factors also formed condensates, including the histone linker protein H1 [55, 56], the H3K9me2/3 ‘writer’ Su(Var)3-9 Homolog 1/2 (SUV39H1/2), and a HP1α-interacting protein, tripartite motif containing 28 (TRIM28) (also called KRAB-associated protein 1, KAP1) [17]. We recently reported that the DNA double strand break (DSB) repair factor, 53BP1 formed liquid condensates at heterochromatin with HP1α in a mutually dependent manner [11, 57]. Loss of HP1α or 53BP1 led to unstable heterochromatin structure and aberrant transcription of repetitive heterochromatic regions, promoting genomic instability [11, 57]. Most importantly, this LLPS function of 53BP1 is independent of its canonical role in DSB repair [11, 57], unveiling a previously unrecognized function of this widely studied gene in genome stability maintenance.

In addition to proteins, the surrounding environment also contributes to the regulation of LLPS at heterochromatin. Liquid phase separation of HP1α at heterochromatin is largely mediated by electrostatic forces as the concentration of HP1α required for droplet formation increases with increasing salt concentration [12]. Hence, condensate formation at heterochromatin is sensitive to the local electrostatic force, redox status, pH, and the property of the water solvation [13]. Further studies revealed how protein modification of HP1α contributes to its LLPS at heterochromatin. Phosphorylation promotes the oligomerization of yeast HP1α, switch mating 6 (Swi6), with histone H3 Lys 9 tri-methylation (H3K9me3) to increase the accessibility of otherwise buried core histone residues in the nucleosome to solvents, enabling the compaction of chromatins into liquid droplets with concomitant increases in the nucleosome concentration [15]. These findings offer new insights into the conceptualization of the assembly and the regulation of heterochromatin.

Another important nuclear event that is impacted by LLPS is the evolutionally conserved DNA damage response, which is crucial for preserving the genome stability. A common feature of the DNA damage response is the formation of distinct foci of proteins at the damage site in the nucleus. The size and the shape of these DNA damage foci resemble those of condensates/inclusion bodies formed through LLPS. Further, since LLPS allows efficient assembly and disassembly of protein complexes in a confined cellular compartment, it has been suggested that DNA damage foci may also undergo LLPS. The prion-like DNA/RNA-binding protein fused in sarcoma (FUS), whose mutation is associated with the motor neuron degenerative disease Amyotrophic Lateral Sclerosis (ALS) through the conversion of FUS-formed liquid droplets into solid aggregates in the neurons, was found to accumulate at DNA damage sites [58], where it mediated the retention of Ku autoantigen 80 (KU80) and the induction of high order of gamma histone H2A X variant (γH2AX) accumulation in a way dependent on the LLPS function of FUS [59]. Several RNA binding proteins that undergo LLPS are also involved in DNA damage response and repair such as splicing factor proline- and glutamine-rich (SFPQ), non-POU domain-containing octamer-binding (NONO), and RNA binding protein 14 (RBM14) [60–63]. Agents like 1,6-hexanediol that are known to disrupt liquid droplets, but not solid gel-like aggregates, reduced the DNA damage response and repair in the presence of chemotherapeutic drugs [59], supporting the role of LLPS in regulating the DNA damage response.

### How LLPS is formed and affected by environmental cues?

At the molecular level, liquid droplet formation is largely driven by the presence of weak and transient intermolecular interactions including nonspecific hydrophobic and electrostatic forces, intermolecular and intramolecular π–π stacking, cation–π interactions, etc. (Fig. 1A) [64, 65]. These interactions are mainly provided by the scaffold protein components that drive the formation of the inclusion bodies with assistance from client molecules that facilitate the condensate formation [6, 38]. Functional groups in a protein (being a scaffold or a client) that can contribute to such intermolecular interactions generally comprise intrinsically disordered regions (IDR) and/or low complexity domains (LCDs). A widely known LCD is the short patches of repetitive peptide sequences such as RGG, FG, FGG, or poly-Qs that mediate protein–protein or protein-RNA interactions and are involved in neurodegenerative disease progression [66, 67]. Each IDR or LCD may contain one or multiple binding points/motifs, or ‘‘stickers’’ [65, 68–71], such as those found in multivalent short linear motifs (SLiMs, like SH3) [9]), oligomerization domains, and even nucleic acids [6, 72–74]. For annotated human genome, about 44% of the encoded proteins contain at least 10% IDR/LCD regions [75, 76]. Hence, “stickers” are widely distributed in the human proteome [73, 74], which can explain why LLPS is so frequently observed for a wide variety of proteins involved in different cellular processes.

LLPS can be formed by the same species of molecules (so called homotypic condensates, like those formed by nucleophosmin 1 (NPM1) [77]) or different molecules (so called heterotypic condensates, like those formed by proteins and RNAs [9, 10]). The sum of the weak interactions provided by these molecules is believed to out compete those between water molecules and the macromolecules, allowing the aggregation of these macromolecules into membraneless condensates in an aqueous solution. The secondary structure of RNAs [78] or the presence of protein posttranslational modifications such as phosphorylation, methylation and SUMOylation, and small molecules may alter (promote, dissolve or a biphasic effect with promoting first following by dissolving [79–83]) LLPS by, for instance, changing the charges and/or other physical properties of the IDRs in these liquid condensates [7]. These multiple modules offer flexibility on the formation/deformation of these liquid condensates in responsive to environmental or physiological stimuli.

### How to measure LLPS in vitro, in cells, and in animal models?

When considering protein aggregates as liquid droplets undergoing phase separation, the physicochemical features of these aggregates in vitro and in cell cultures need to be determined. Here, we will briefly summarize commonly used methods to characterize and understand LLPS, as detailed description can be found in recently published excellent reviews [6, 8, 39].

The first key determinant for liquid condensate formation is to run in vitro assembly assay using purified soluble proteins, and nucleic acids if needed. By doing so, it can allow (1) the observation of liquid droplet formation under bright field microscopy. If proteins are labeled with fluorescence tags or dyes, the liquid condensates can be visualized under fluorescence microscopy. (2) The determination of the saturation concentration (C_sat_), where when C < C_sat_, the protein is diffuse in solution, whereas when C > Csat, the droplets could form. The determination of the C_sat_ will distinguish liquid droplet formation from protein dimerization or oligomerization, as the latter usually will not change their high-order assembly even when the concentrations or environmental factors are altered. However, for heterotypic condensates assembled in cells such as those formed by proteins and RNAs, the Csat may vary and phase separation does not depend on a fixed Csat [84]. (3) The measurement of the turbidity of condensates due to their effects on inducing light scattering, although it is not able to distinguish liquid droplets from gel or solid condensates. (4) Fluorescence recovery after photobleaching (FRAP) has been routinely used in vitro and in cells to determine the mobility and diffusion coefficient of condensates formed in test tubes or aggregates/inclusion bodies formed inside cells. To determine the particle diffusion more precisely, fluorescence correlation spectroscopy (FCS) can be performed, in which fluorescence intensity fluctuation within a small volume due to molecule diffusion will be measured over time to obtain detailed information about the size, the dynamics, and the concentrations of fluorescent particles within. Unlike droplets formed in vitro, in vivo cell culture assessment of liquid droplets is challenging due largely to much lower concentrations of scaffold (or client) proteins. FRAP has been suggested as the standard method for measuring LLPS in cell cultures, but requiring expression of fluorescence tagged proteins. Hence, orthogonal assays should be performed to validate the results obtained from FRAP, such as live cell imaging and treatment with chemicals like 1,6-hexanediol to disrupt the weak inter-molecular hydrophobic interactions [21]. In addition, overexpression of fluorescently tagged proteins can be used to determine if there is a protein concentration-dependent liquid condensate formation in cells and whether such condensates will be affected by changing the culture conditions (raising the temperature or adding LLPS inhibiting agents) or the protein’s posttranslational modifications. Further, fluorescently tagging the genomic locus by genome editing tools can allow the evaluation of phase separation of endogenous proteins.

One point that is worth mentioning is the use of crowding agent such as polyethylene glycol, dextran or ficoll in the in vitro LLPS assembly assay, as these agents can promote or at least enhance condensate formation for proteins that otherwise did not form aggregates [85]. Hence, it is recommended to limit the use of crowding agent when conducting in vitro liquid droplet formation assays [6]. This is exemplified by the different outcomes between what we recently reported and previously published in assessing 53BP1 liquid droplet formation in vitro. Previously, a crowding agent (Ficoll 400) was added to the in vitro liquid droplet assembly experiment, in which the C-terminus of 53BP1 did form liquid droplets by itself at a concenration of ~ 2 μM in the presence of 12.5% Ficoll [86]. However, we did not observe liquid condensate formation in vitro by the same 53BP1 fragment without any crowding agents even when its concentration was raised to 10 μM [11]. Yet, when purified human HP1α proteins were included, the 53BP1 fragment and HP1α quickly formed liquid droplets in vitro without any crowding agents. Hence, when using crowding agents, additional assays should be conducted to validate the liquid condensate formation capability of the protein/peptide of interest.

Currently, tools and techniques are generally lacking to assess LLPS accurately and quantitatively in animal models except the detection of protein condensates or aggregates by fluorescence tag or antibodies. Nonetheless, a variety of animal models have been used to assess LLPS under physiological setting or to recapitulate the onset and/or the progression of diseases caused by abnormal condensate formation. *C. elegans* embryos facilitated our understanding of germline P granules at one-cell stage [2] and PGL granule formation by heat shock during embryogenesis [87]. Neuron-specific expression of FUS mutants found in human ALS/FTD patients resulted in amyloid formation in *C. elegans* and the animals demonstrated age-dependent motor function impairment and shortened lifespan; further, the neurotoxicity is associated with the ability of mutant FUS to form irreversible aggregates [88]. Familial ALS patients with a FUS mutation (R495X) demonstrated more severe phenotype and shortened survival, and injection of such mutant into zebrafish embryo showed cytoplasmic accumulation of the mutant proteins in the spinal cord and demonstrated impaired stress granule response [89]. Transgenic *Drosophila* animals expressing a prion-like mutant of human heterogeneous nuclear ribonucleoprotein A1/2 (hnRNPA1/2), which form inclusion bodies in the cytoplasm, resulted in muscle degeneration [90]. Budding yeast Ataxin-2 (also called Pbp1) specifically responds to mitochondrial respiration to inhibit mTORC1 through phase separation, leading to autophagy activation [91]. In a *Kras* and *Trp53* mutant lung adenocarcinoma mouse model, the Hippo pathway effector Yes-associated protein (YAP) formed condensates, which are associated with resistance to immune checkpoint inhibitors; accordingly, cancer cells expressing YAP phase separation defective mutants demonstrated strong response to the checkpoint inhibitors in mice [92]. Overall, compared with the vast majority of in vitro and cell culture models, animal model studies are needed to validate the functional significance of LLPS in biology, physiology, and pathophysiology.

### Roles of LLPS in human health and disease

Properly regulated liquid condensate formation provides a unique means to control cellular function that cannot be easily recapitulated by the individual component, especially when responding to internal or external stimuli. Unfortunately, abnormalities in this process could lead to unwanted effects with a particular concern on the conversion from the flexible liquid droplets into rigid and probably irreversible gel-like, or worse, solid-like aggregates, which will abolish the ability of LLPS to rapidly assembly and disassembly upon environmental changes (Fig. 1B, i-iii). Such a detrimental conversion is best exemplified by the progression of neurodegenerative diseases such as ALS, Frontotemporal Dementia (FTD), Alzheimer’s diseases (AD), Parkinson’s diseases (PD), Inclusion Body Myopathy (IBM), Multisystem Proteinopathy (MSP), etc., whose progression is accompanied with gel-like aggregate formation of pathogenic proteins.

For instance, stress granule proteins including nuclear ribonucleoproteins (hnRNPs), TIA-1, TDP-43, tubulin associated unit (Tau), FUS, α-synuclein, and those involved in the regulation of stress granules (e.g., vasolin-containing protein VCP/P97 and Profilin 1) tend to assemble into amyloid-like fibrils or nucleate fibrous aggregates instead of forming liquid condensates during aging, which is considered a common feature of these diseases that may facilitate, promote or at least correlate with the disease progression [18, 58, 88, 90, 93–100]. In the presence of disease-associating mutations, the irreversible transition from the liquid-like condensates to gel-like fibrils could be accelerated, contributing to neurodegenerative disease progression [18, 58, 89, 90, 99, 101–108] (Fig. 1B). Evidence that supports abnormal LLPS to disease progression comes from the observation that those pathogenic mutations fall within the IDRs or domains of the proteins that are crucial for their LLPS seen in ALS [90], FTD [109, 110], PD [111], AD [112], or cancers [113]. In addition to mutations, posttranslational translation modifications such as phosphorylation, acetylation, SUMOylation, ubiquitination, etc., could also modulate the capability of proteins to form pathological condensates and affecting the disease progression [111, 112, 114, 115]. The detailed molecular mechanisms underlying the conversion of liquid condensates into gel- or solid-like aggregates remain unknown and could be a case dependent manner; however, it is tempting to speculate that mutations or acquired posttranslational modifications of proteins (especially on the scaffold components of the droplets) alter or disrupt the equilibrium of phase separation in the condensates, leading to irreversible formation of pathological aggregates.

In addition, liquid droplet formation and phase separation have been observed during male germ cell differentiation [116], cancer progression [117–119], inflammation [120], plant immunity [121], and infection by virus including the Sars-Cov-2 virus that caused the Coronavirus disease 2019 (COVID-19) pandemic and parasites [122–127]. For instance, mutant P53 proteins can form amyloid-like aggregates, which lost its tumor suppressing function [128] or contributed to cancer therapy resistance [129–131]. These findings further support the importance of LLPS in pathophysiology and disease progression.

### Targeting LLPS in disease treatment

Given the increasing importance of LLPS in disease development and progression, targeting LLPS represents a promising strategy to correct/ameliorate pathophysiological disorders. Many attempts have been made by the scientific community and a large body of studies have revealed various types of small molecules in mediating LLPS, condensate or aggregate formation. For instance, digitoxin reduced stress granule formation independent of eIF2α phosphorylation [132], indicating that this compound acts on the stress granule formation, but not upstream regulatory events. Ribosomal inhibitors such as anisomycin and neymycin reduced stress granule formation [132], similar to the known ribosomal E-site binding molecule, cycloheximide. Actinomycin D, an RNA polymerase I inhibitor, reduced the stiffness of and changed the proteome in the nucleoli [133, 134]. ATP can function as a hydrotrope to promote protein solubility and therefore inhibits liquid condensate formation; in contrast, depletion of ATP increased the viscosity of nucleoli [4] and promoted LLPS formation of stress granules [135, 136]. 1,6-hexanediol, an alcohol derivative, disrupts the weak inter-molecular hydrophobic interactions probably through altering hydrogen bonds, and has been used at a range of 1–10% (vol/vol) concentration to distinguish liquid droplets from solid gel-like assemblies in vitro and in living cells [21, 137, 138]. Lipoamide came out from a compound screen to reduce the formation of cytoplasmic FUS stress granules induced by arsenate [139]; further, lipoamide mitigated stress granule formation by mitochondrial electron transport chain inhibition, or hyperosmotic stress, but had no effect on those induced by heat shock or glycolysis inhibition, suggesting that this compound does not just simply function as an antioxidant, but instead can inhibit various liquid condensate formation. Interestingly, lipoamide selectively inhibits stress granule formation, but not those of Cajal body, nucleoli or DNA damage foci [139]. Mitoxantrone, a heterotricyclic compound, reduced condensate formation of stress granules, Cajal body, nucleoli or DNA damage foci [139], which was independently confirmed by another study [132].

Neurodegenerative diseases have been shown to be a typical example of pathological LLPS with excessive condensate formation. A number of small molecules like mitoxantrone and lipoamide that could disrupt cation–π interactions and therefore alter the partition process in the liquid condensates have been shown to prevent FUS or stress granule proteins from undergoing LLPS [65, 132, 139]. Congo red, a dye commonly used to determine amyloid aggregates in patients’ brain [140], also induced biphasic LLPS of TDP-43/LCD and FUS/LCD proteins likely due to the presence of both highly hydrophobic moiety (naphthalene) and negatively charged groups [80]. A synthetic curcumin derivative C1 was reported to inhibit aggregate and filament formation of tau, whose pathological aggregation is associated with neurodegenerative disorders like AD [141]. Cellular assays showed a protective effect of curcumin C1 against tau oligomer-induced neuron cell death [141]. Planar moiety-bearing compounds such as quinacrine, doxorubicin and daunorubicin reduced the number and the size of stress granules formed in the presence of various types of stress, likely through intercalating with nucleic acids presented in the stress granules [132]. Doxorubicin and quinacrine altered LLPS of RNA-containing condensates [34, 142] and prion-like protein aggregates [143], which may demonstrate a novel role in treating neurodegenerative diseases in addition to being a widely used chemotherapeutic agent. Poly (ADP-ribose) polymerase 1 (PARP1), a key player in DNA damage repair, especially for single strand DNA break repair [144], was reported to contribute to a LLPS environment at DNA damage site through protein PARylation [145]. Other than DNA damage, PARylation mediates the nucleation of prion-like condensates formed by TDP-43 and FUS [145, 146]. Hence, agents that block LLPS resulting from protein PARylation may enhance the effect of PARP inhibitors in cancer therapy or prevent pathogenic amyloid aggregation in neurodegenerative diseases [147].

Some compounds demonstrate opposite effects on LLPS in a concentration dependent manner. 4,4’-dianilino-1,1’-binaphthyl-5,5’-disulfonic acid (bis-ANS), a fluorescent molecule that was initially applied to mark exposed hydrophobic patches in proteins and to detect protein aggregate formation [148, 149], is a biphasic agent that could induce (at lower concentration) or prevent (at higher concentration) LLPS formed by purified TDP-43/LCD or FUS/LCD proteins [80]. This biphasic LLPS assembly/disassembly is likely determined by the structure of bis-ANS, which may engage in ionic charge−charge or cation−π interactions with charged groups on proteins [79]. A similar biphasic LLPS formation was observed for TDP-43/LCD in the presence of poly(A) to mimic the presence of RNA. However, such biphasic effect has not been demonstrated in cell cultures, and a possible explanation is that this compound (or compound type) demonstrates toxicity to cells at high concentrations that are needed to form liquid droplets.

Other than neurodegenerative diseases, cancer has been increasingly reported to be associated with LLPS; therefore, targeting LLPS could become a new frontline in the anticancer drug development. A small molecule screen identified a 3-phenylquinazolinone like compound isFSP1 as a potent inhibitor of the ferroptosis suppressor protein-1 (FSP1). Interestingly, icFSP1 induces phase separation and condensate formation of FSP1, through which it synergizes with inhibition of cysteine glutathione peroxidase 4 (GPX4) to enhance ferroptosis of cancer cells and to suppress tumor growth in mice [150]. On the other hand, a small molecule ET516 was identified through a phenotypic screen to disrupt phase separation of androgen receptors, overcoming drug resistance of mutant androgen receptor expressing prostate cancers [151]. The tumor suppressor protein P53 could form aggregates and the disease-relevant mutants accelerated the process to form pathogenic amyloid-like amorphous aggregates, oligomers, and even fibrils that blocked the transcriptional function of P53 [128, 152–156], which may also contribute to therapy resistance [129–131]. Hence, disruption of the condensate formation by mutant P53 proteins represent a useful strategy to treat cancers that bear *TP53* mutations [153, 157–159]. Encouraging data are emerging to support this idea. For instance, aminothiazole-type compounds BAY249716 and BAY1892005 bind to and stabilize P53 proteins, resulting in a reduction in nuclear puncta formation by overexpressed P53 mutant proteins accompanied with the rescue of wild type P53 activities [157]. Small molecule PRIMA-1 that covalently modifies the Cys124/135/141 of P53 inhibited P53 aggregation, and a cell-permeable peptide ReACp53 derived from the distorted region of P53 (252–268 amino acids) inhibited the amyloid-like aggregate formation by the pathogenic P53^R248Q^ mutant in primary high grade serous ovarian carcinoma cells, rescuing wild type P53 function (i.e., cell cycle arrest and apoptosis induction) in ovarian cancer [158]. However, whether the real in vivo target of this kind of peptide is indeed the aggregated P53 mutant protein remains to be determined [159]. The aggregates formed by the P53^Y220C^ mutant can also be suppressed by a ligand that binds to the Y220C mutation-induced cavity of P53 [160].

**Fig. 2:**
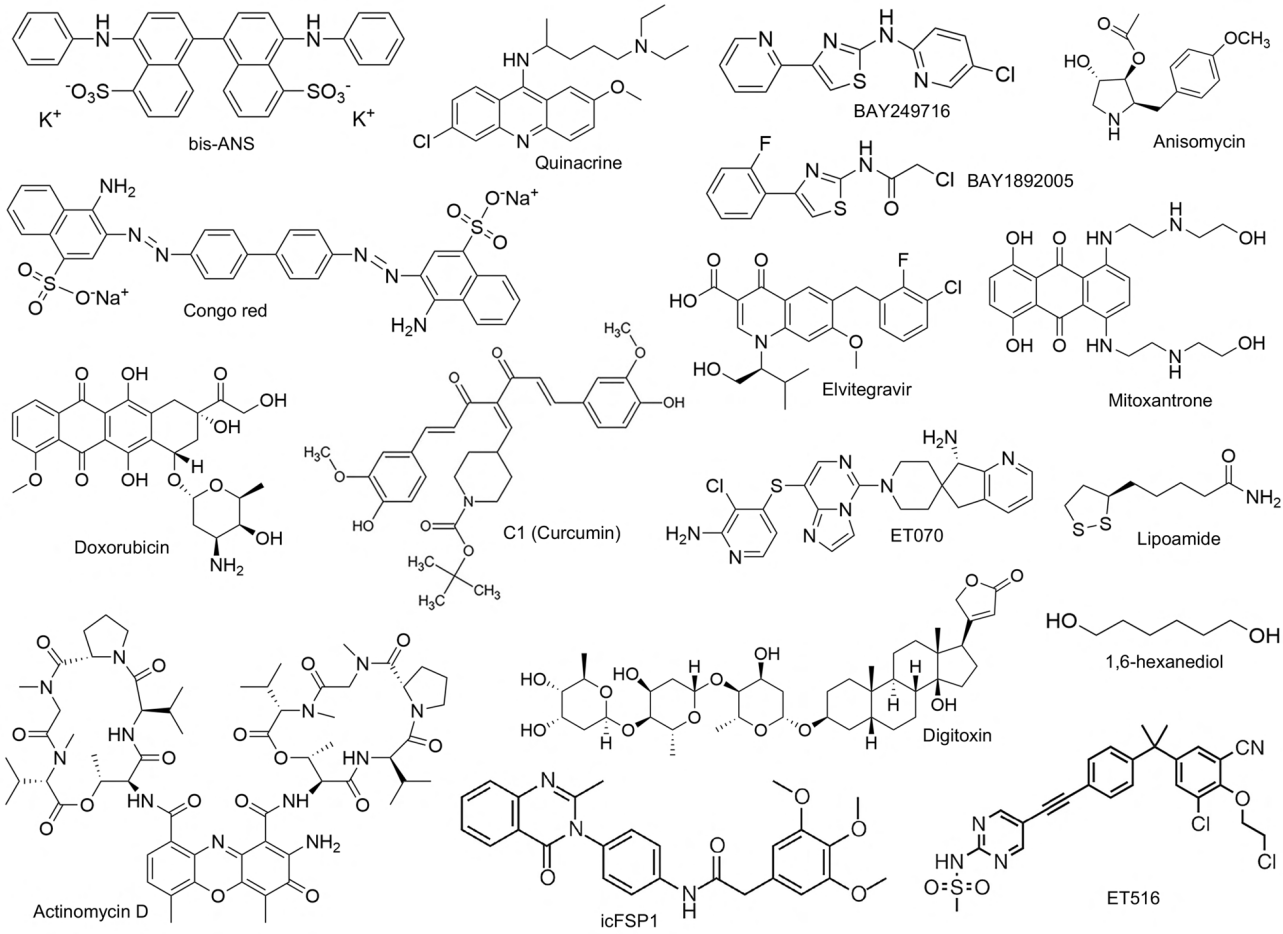
Structures of reported chemicals that showed activities in altering LLPS

As presented in Table 2 and Fig. 2, these compounds include natural products and their derivatives such as antibiotics, cardiac glycoside, fluorescence probes, heterocycle compounds, lipophilic or aliphatic compounds, etc. We noticed that these compounds demonstrate little common chemical properties. Nonetheless, many of them contain unsaturated benzene or benzene-like rings, representing a potential starting point for future medicinal chemistry modification and small molecule screen.

**Table 2:** Chemicals that alter condensate formation

Name	Formula	Potential LLPS altering function	References
Actinomycin D	C_62_H_86_N_12_O_16_	An RNA polymerase I inhibitor, reduced the stiffness of and changed the proteome in the nucleoli	[133, 134]
ATP	C_10_H_16_N_5_O_13_P_3_	A hydrotrope that promotes protein solubility and inhibits liquid condensate formation. Depletion of ATP increased the viscosity of nucleoli and promoted LLPS formation of stress granules	[4, 135, 136]
1,6-hexanediol	C_6_H_14_O_2_	The most widely used chemical to inhibit liquid condensate formation by disrupting weak inter-molecular hydrophobic interactions	[21, 137, 138]
BAY249716 BAY1892005	C_13_H_9_N_4_SCl C_11_H_8_ClFN_2_OS	Inhibited condensate formation by mutant P53 proteins	[157]
PRIMA-1	C_9_H_15_NO_3_	Inhibited P53 aggregate formation by covalently modifying P53	[158]
Lipoamide	C_8_H_15_NOS_2_	Inhibited FUS stress granule formation by arsenate	[139]
Mitoxantrone	C_22_H_28_N_4_O_6_	Reduced condensate formation by stress granules, Cajal body, nucleoli or DNA damage foci	[65, 132, 139]
bis-ANS	C_32_H_22_K_2_N_2_O_6_S_2_	Promoted (low conc) or reduced (high conc) FUS and TDP43 condensates	[80]
Congo red	C_32_H_22_N_6_Na_2_O_6_S_2_	Promoted (low conc) or reduced (high conc) FUS and TDP43 condensates	[80, 140]
Quinacrine Doxorubicin Daunorubicin	C_23_H_30_ClN_3_O C_27_H_29_NO_11_ C_27_H_29_NO_10_	Reduced condensate formation of stress granules	[34, 132, 142, 143]
Digitoxin	C_41_H_64_O_13_	Reduced stress granule formation	[132]
Anisomycin Neymycin	C_14_H_19_NO_4_ C_23_H_46_N_6_O_13_	Reduced stress granule formation	[132]
Elvitegravir	C_23_H_23_ClFNO_5_	Inhibited SRC-1 condensates	[174]
ET070	C_23_H_20_ClN_8_S	SHP2 allosteric inhibitor, reduced condensate of pathogenic SHP2	[173]
Curcumin C1	C_22_H_37_NO_8_	Inhibited tau aggregation and filament formation	[141]
icFSP1	C_26_H_25_N_3_O_5_	Induced condensate formation and phase separation of FSP1 to induce ferroptosis	[150]
ET516	C_25_H_22_N_4_Cl_2_SO_3_	Inhibited phase separation of androgen receptor mutants to inhibit prostate cancer growth	[151]

In all, targeting LLPS represents a burgeoning field that has great promise in treating various human diseases. We expect to see more reports of high throughput drug screening that identify novel compounds altering LLPS [161]. This kind of studies will increase the pool of chemicals that affect the LLPS process, allowing medicinal chemists to better derive chemical backbones or moieties that are important for regulating LLPS and ultimately disease targeting.

### Challenges in LLPS research and potential solutions

Through years of extensive research, we have gained a good understanding about how LLPS is formed, what biological processes it regulates, and how it may contribute to disease progression. Tools and methods that can determine LLPS, especially its physicochemical properties in vitro, are available, although the ones that can confirm the presence of LLPS for endogenous proteins in vivo are still lacking. However, some major challenges remain since the LLPS concept was introduced to the biology field.

First, understanding the true functional relevance of LLPS in biology is perhaps the first and foremost critical issue. Many reports focused on the liquid droplet formation in vitro by purified proteins, with some also demonstrating the presence of liquid droplets/condensates formed inside cells but largely, if not entirely, by overexpressed proteins or the IDR fragments. A couple of reasons can be considered for why endogenous proteins are difficult to study. (1) The concentration of endogenous proteins is often much lower than purified proteins in vitro, making them difficult to form measurable condensates under physiological conditions. (2) Endogenous proteins are not suitable for LLPS detection using methods that are currently available in a live cell context. Engineering a fluorescence tag to endogenous proteins by such as clustered regularly interspaced short palindromic repeats (CRISPR)/Cas and then detecting the LLPS properties of tagged proteins in cells can solve, at least partially, this issue, especially for those that can express at relatively high levels in cells.

Although an increasing number of report shows functional importance of LLPS by certain genes, for instance, germ cell differentiation by LLPS of Fragile X messenger ribonucleoprotein 1 autosomal homolog 1 (FXR1) [116], there is a general lack of strong in vivo evidence showing that disrupting LLPS formation results in the loss of cell fitness or developmental defects, which has cast doubt about the true biological relevance of these liquid condensates, or whether these condensates or fibrillar aggregates are causative in pathology. Apparently, studies are needed to show a causative role of aggregate accumulation from aberrant LLPS in pathophysiology or disease initiation and/or progression, which may rely on the advent of animal models that can recapitulate the disease progression in a manner dependent on LLPS defects.

Second, due to the dynamic nature and the fragility of the membraneless liquid droplets, it is extremely difficult to identify and determine protein components in these condensates formed inside cells. Although nucleoli seemed to tolerate cellular fraction and can be isolated for protein identification, this is likely a special case as nucleoli may have as-yet unidentified features that make them more stable than most liquid condensates. For instance, intact nucleoli survived sonication and sucrose density centrifugation and were isolated from HeLa cells, which led to the identification of 271 proteins in it [162]. However, this approach was apparently inefficient as it is believed that nucleoli contain more than 4000 proteins [163], suggesting that the isolation procedure might have partially damaged the condensate integrity and therefore lost most of the components. Apparently, the commonly used cell lysis or even cellular membrane breakdown methods are likely going to disrupt these liquid inclusion bodies, especially those formed in the absence of any cellular structural networks, making the isolation of components in these condensates from the surrounding cellular aqueous solution challenging, if not insurmountable, by regular extraction methods.

To circumvent this issue, introducing a cross linking step before the pulldown of a bait protein component in a specific type of liquid condensate seems to be reasonable (Fig. 1C). In this regard, the latest pulldown approaches such as the proximity labeling derived BioID or TurboID is attractive. However, this biotin-derived techniques is limited in attracting proteins that are within ~ 10 nm range of the bait [164], which apparently does not meet the requirement for proteins in liquid droplets that have a diameter ranging from nm to μm, like the ones formed by 53BP1 [11]. Nonetheless, the idea of crosslinking should stabilize the weak interaction among proteins (or even nucleic acids) in the liquid droplets, facilitating their isolation, identification, and functional characterization (Fig. 1C). This strategy is probably more appropriate for LLPS occurring on structural networks, such as those on cytoskeleton and chromatin, as these structures can provide an additional layer of support for the retention of liquid condensates, making them more resistant to cell lysis and therefore allowing easier isolation of protein components for mass spectrometric identification. For condensates formed in soluble cytoplasm or nuclei, perhaps we have to find ways to solidify these condensates to make them resistant to cell lysis, allowing their isolation based on the particle densities using ultra centrifugation. To more precisely isolate LLPS proteins, introducing a biotin-like tag to endogenous proteins that are also the scaffold proteins of the liquid droplets probably allows more specific crosslinking of proteins in the liquid droplets and therefore the stabilization of such droplets than a general crosslinking approach. In all, methods that can specifically stabilize the protein–protein interaction in a liquid droplet-of-interest are desired to move the field forward.

Third, while we have identified a number of compounds that can interfere with the LLPS or condensate formation process, whether they can be truly developed into drugs remains unknown. Clearly, more efforts are needed to develop efficient assays that can screen efficacious compounds with drug-like properties. Unlike conventional drug targets such as protein kinases, G-protein coupled receptors, and cellular membrane channels that have distinct and clear pockets to allow small molecules to bind, the disordered regions, which are key for assembling liquid droplets/condensates in LLPS, are generally considered to be undruggable due to the lack of stable chemical binding affinity [165–168]. Small molecules that can target partially folded regions are limited [169], although they are not exactly targeting IDRs in liquid droplets. In addition, the weak and transient intermolecular interactions in the dynamic liquid droplets probably won’t serve as a stable platform to attract small chemicals to bind. Further, it seems difficult to find general chemical inhibitors that can disrupt LLPS formed by different proteins while demonstrating drugability, which is supported by the finding that diverse class of compounds (in terms of their structural stereotypes and reported cellular activities) were found to affect the stress granule formation induced by oxidative stress [132] (Table 2). The frequently used 1,6-hexanediol is considered an agent that can inhibit the formation of liquid droplets, but not solid aggregates, which may seem to be a general LLPS inhibitor. However, the exact mechanism by which this chemical disrupts LLPS remains unclear. A recent study showed that this chemical broadly inhibits protein kinases and phosphatases [170], indicating high non-selectivity, which is also consistent with its strong toxicity, precluding it from being a therapeutic agent.

To overcome these challenges, alternative strategies have been actively developed. For instance, approaches were developed to target specific liquid condensates formed by known targetable proteins like kinases and those with fragments of well-organized structures, especially when these molecules are the scaffolds of the liquid condensates [171, 172]. Consistent with this idea, an allosteric inhibitor of Src homology 2 domain containing phosphatase 2 (SHP2), ET070 abolished liquid condensate formation by disease associated SHP2 mutants and suppressed the downstream signaling activation [173]. Similarly, an anti-HIV chemical, elvitegravir, blocked liquid condensates formed by the histone acetyltransferase steroid receptor coactivator 1 (SRC-1), which suppressed the transcriptional activity of the oncogenic Yes-associated protein (YAP) [174].

Unlike physiological LLPS where molecules are accumulated through weak, transient, and reversible intermolecular interactions, pathogenic phase separation likely demonstrates enhanced and probably irreversible intermolecular interactions to allow the aggregation of gel- or solid fibril-like structures (Fig. 1C). These much more stable structures in theory can be appropriate targets for small molecules. In addition, such solid structures may favor the targeting of monoclonal antibodies that specifically recognize their structures or shapes that are not seen in normal endogenous proteins (Fig. 1C), promoting a long lasting cure. Diseases that are believed to be affected most by protein aggregation are neurodegenerative diseases including AD, PD, ALS, etc*.*, through the formation of prion-like aggregates by amyloidogenic proteins. Therefore, strategies that can revert or mitigate these aggregates hold the promise of ameliorating or improving the disease prognosis and patient outcome.

## Conclusions

1. Liquid–liquid phase separation (LLPS) involves the assembly of membraneless organelles by macromolecules (mainly proteins but also nucleic acids like RNAs).
2. The forces that promote LLPS comprise weak intermolecular interactions provided by disordered peptide regions or low complexity domains commonly found in proteins and RNAs.
3. While normal LLPS represents an emerging cellular process that regulates biology and physiology, uncontrolled or pathogenic LLPS promotes the progression of various human diseases, particularly the progressive neuronal disorders.
4. LLPS offers unique opportunities in finding new therapies to target human diseases caused by abnormal or pathogenic LLPS.
5. Innovative strategies are needed to identify the protein components in liquid droplets, as well as molecules (being a small chemical or biological agent like antibodies) that can target LLPS for disease management.

## Data Availability

Not applicable.

## Abbreviations

53BP1: TP53 binding protein 1
AD: Alzheimer’s diseases
ALS: Amyotrophic lateral sclerosis
cAMP: 3’,5’-Cyclic adenosine monophosphate
cGAS: Cyclic GMP-AMP synthase
CRISPR: Clustered regularly interspaced short palindromic repeats
C_sat_: Saturation concentration
COVID-19: Coronavirus disease 2019
DSB: DNA double strand break
FCS: Fluorescence correlation spectroscopy
FRAP: Fluorescence recovery after photobleaching
FSP1: Ferroptosis suppressor protein-1
FUS: Fused in sarcoma
FXR: Fragile X messenger ribonucleoprotein 1 autosomal homolog 1
GPX4: Cysteine glutathione peroxidase 4
γH2AX: Gamma histone H2A X variant
H3K9me3: Histone H3 Lys 9 tri-methylation
HP1α: Heterochromatin protein 1α
hnRNP: Human nuclear ribonucleoprotein
IBM: Inclusion body myopathy
IDR: Intrinsically disordered region
KAP1: KRAB-associated protein 1
KU80: Ku autoantigen 80
LCD: Low complexity domain
LLPS: Liquid–liquid phase separation
NONO: Non-POU domain-containing octamer-binding
MSP: Multisystem proteinopathy
NPM1: Nucleophosmin 1
PD: Parkinson’s diseases
PARP1: Poly (ADP-ribose) polymerase 1
PKA: Protein kinase A
RBM14: RNA binding protein 14
RNP: RNA ribonucleoprotein
SFPQ: Splicing factor proline- and glutamine-rich
SHP2: Src homology 2 domain containing phosphatase 2
SLiM: Short linear motif
SRC-1: Steroid receptor coactivator 1
SUV39H1: Su(Var)3–9 Homolog 1
Swi6: Switch mating gene 6
Tau: Tubulin associated unit
TCR: T-cell receptor
TDP-43: Transactive response DNA binding protein of 43 kDa
TRIM28: Tripartite motif containing 28
VCP/P97: Vasolin-containing protein
YAP: Yes-associated protein
